# Comparison of patients in three different rehabilitation settings after knee or hip arthroplasty: a natural observational, prospective study

**DOI:** 10.1186/s12891-015-0780-2

**Published:** 2015-10-24

**Authors:** T. Benz, F. Angst, P. Oesch, R. Hilfiker, S. Lehmann, C. Mueller Mebes, E. Kramer, ML Verra

**Affiliations:** Research Department, RehaClinic, Bad Zurzach, Switzerland; Kliniken Valens, Rehabilitationszentrum Valens, Valens, Switzerland; School of Health Sciences, HES-SO Valais-Wallis, Leukerbad, Switzerland; Department of Physiotherapy, Bern University Hospital, Bern, Switzerland; Klinik Adelheid, Unterägeri, Switzerland

**Keywords:** Hip and knee arthroplasty, Inpatient rehabilitation, Convalescence center, Ambulatory treatment

## Abstract

**Background:**

Patients after primary hip or knee replacement surgery can benefit from postoperative treatment in terms of improvement of independence in ambulation, transfers, range of motion and muscle strength. After discharge from hospital, patients are referred to different treatment destination and modalities: intensive inpatient rehabilitation (IR), cure (medically prescribed stay at a convalescence center), or ambulatory treatment (AT) at home. The purpose of this study was to 1) measure functional health (primary outcome) and function relevant factors in patients with hip or knee arthroplasty and to compare them in relation to three postoperative management strategies: AT, Cure and IR and 2) compare the post-operative changes in patient’s health status (between preoperative and the 6 month follow-up) for three rehabilitation settings.

**Methods:**

Natural observational, prospective two-center study with follow-up. Sociodemographic data and functional mobility tests, Timed Up and Go (TUG) and Iowa Level of Assistance Scale (ILOAS) of 201 patients were analysed before arthroplasty and at the end of acute hospital stay (mean duration of stay: 9.7 days +/− 3.9). Changes in health state were measured with the Western Ontario and McMaster Universities Osteoarthritis Index (WOMAC) before and 6 months after arthroplasty.

**Results:**

Compared to patients referred for IR and Cure, patients referred for AT were significantly younger and less comorbid. Patients admitted to IR had the highest functional disability before arthroplasty. Before rehabilitation, mean TUG was 40.0 s in the IR group, 33.9 s in the Cure group, and 27.5 s in the AT group, and corresponding mean ILOAS was 16.0, 13.0 and 12.2 (50.0 = worst). At the 6 months follow-up, the corresponding effect sizes of the WOMAC global score were 1.32, 1.87, and 1.51 (>0 means improvement).

**Conclusions:**

Age, comorbidity and functional disability are associated with referral for intensive inpatient rehabilitation after hip or knee arthroplasty and partly affect health changes after rehabilitation.

## Background

Joint replacement surgery is a routine treatment for advanced osteoarthritis of the hip and knee. The primary treatment goal is the restoration of independence in the activities of daily life by reduction of pain and disability [1]. Arthroplasty is one management option recommended by the European League against Rheumatism (EULAR) alongside medication, exercise therapy and patient education [2, 3]. Health-related quality of life was observed to be improved after arthroplasties of the hip and knee [4–7].

From 2005 to 2008 the number of implanted hip and knee arthroplasties in Switzerland increased from 238 to 262, and from 140 to 177 respectively per 100,000 inhabitants per year [8]. In 2011, 306 hip and 205 knee replacement surgeries per 100,000 citizens were performed in Switzerland [9].

Postoperative management directly after hip or knee arthroplasty in the acute hospital includes physiotherapy which is, in most countries, accepted as standard treatment. The main focus of physiotherapeutic treatment is on mobilization on the first or second postoperative day, walking instruction with crutches according to the weight bearing restrictions depending on the fixation of the implants and on individual surgeons’ preferences, as well as minimization of complications (e.g. wound infection, deep vein thrombosis or pulmonary embolism) [1, 10–12]. The prevention of hip dislocation and the focused improvement on range of motion after knee arthroplasty are additional, joint-specific aims of physiotherapeutic treatment in hospital [10, 12].

In Switzerland, physiotherapeutic and medical treatment generally continue after discharge from the acute care hospital to different inpatient or outpatient settings. Patients may be discharged home with ambulatory medical and physiotherapeutic treatment (AT). This outpatient treatment may take place, according to the patient’s preference, at home (home-based), in an outpatient treatment setting or in an outpatient clinic. More intense treatment options include the medically prescribed stay at a convalescence center (Cure) or inpatient rehabilitation (IR) in a specialized orthopedic rehabilitation clinic providing 24 h of medical care.

The decision where to refer the patient for post-hospital care is made by the surgeon in consultation with the patient. Insurance companies are asked to grant payment for IR or Cure based on a detailed description of the patient’s medical record by the surgeon. Age, comorbidities, social situation at home and dependence on nursing support in the hospital are supposed to be taken into consideration and included in the report for the insurance company. Immobility and lack of social support at home may require more intensive inhouse treatment. Whether these decision-making factors are really applied in clinical reality is unclear and empirically not proven. However, there are no evidence-based criteria such as a threshold score from a functional assessment that facilitates the decision-making process with regard to the future direction of post-hospital care. The post-acute phase of rehabilitation is less well documented and researched than the acute care phase, but includes therapeutic exercise (joint mobilization, muscle strengthening), transfer and gait training as well as instruction of the activities of daily living [11, 12].

The first aim of this study was to measure functional health (primary outcome) and function relevant factors in patients with hip or knee arthroplasty and to compare them in relation to three postoperative management strategies: AT, Cure and IR. The first hypothesis was that older, more immobile patients with a higher number of comorbidities who are living alone are more likely to be referred to IR than to Cure or AT. Further, we analysed the changes in health observed after the three different rehabilitation settings. The second hypothesis was that higher score changes were expected in the IR group followed by the Cure group and the lowest score changes in the AT group. In other words, therapy intensity was expected to be positively associated to the size of the score changes. To the best of our knowledge, there are no studies that have analysed the relationship between these rehabilitation settings and the change of health state in patients with hip or knee arthroplasty.

## Methods

### Patients

Patients were recruited at the Cantonal Hospital of St. Gallen (KSSG) and at the Inselspital (ISB), Bern University Hospital in Berne, Switzerland. In the Departments of Orthopedics, patients with hip or knee osteoarthritis who were referred to the hospitals for primary hip or knee arthroplasty by their family physician or rheumatologist were consecutively invited to participate in the study. Patients of 50 years of age or older were included if they were scheduled for a primary total or partial arthroplasty. Exclusion criteria included insufficient German language skills to complete the questionnaires and refusal to participate in the study. During follow-up patients were excluded it they refused further participation, did not return the questionnaires or if their questionnaires were incomplete according to the missing rules of the Western Ontario and McMaster Universities Osteoarthritis Index (WOMAC) [13].

For sample size calculation of differences of score changes between two groups, a t-test formula was used setting type one error to alpha = 0.05 and type two error, beta = 0.20 (power = 0.80). We assumed a standard deviation of 20 score points of the score differences between baseline and follow-up for each group and difference of the score differences (each between baseline and follow-up) of 8 score points. This corresponds to a standardized mean difference of 0.40 which is the size of an effect that has been determined as minimally clinically important (perceptive for the patients) for the WOMAC global score [14]. This leads to a minimal total sample size for the two examined groups of *n* = 100 for each of the pairwise group comparisons.

Written informed consent was obtained from all participants. The cantonal ethic committees of Bern and St. Gallen, to which the study protocol was submitted, classified this study protocol as “no risk” and confirmed that no project approval needed to be granted.

### Intervention

In this natural observational, prospective two-center study, pre- and postoperative treatments as well as surgery were conducted according to the standard treatment protocol of the Cantonal Hospital of St. Gallen (KSSG) and the Inselspital (ISB), Bern University Hospital in Bern, Switzerland. These protocols included preoperative treatment in the hospital with information about surgery and physiotherapeutic instruction about postoperative proceedings including instruction about postoperative prevention of pneumonia, technique of mobilization and walking with crutches.

Post-hospital rehabilitative treatment after hip or knee arthroplasty included IR, Cure and AT. These rehabilitative interventions optimize surgical outcomes by reducing pain, improving strength and function and by minimization of complications. There is insufficient scientific evidence about the precise rehabilitative and therapeutic techniques that should be applied. In general, treatment is led by the type and severity of the symptoms presented at the medical examination at admission to rehabilitation as well as by the recommendations of the surgeon. Therefore, the individual treatment in this naturalistic study was not standardized or accounted for in the analysis.

The intensity of treatment was defined by the type of rehabilitation prescribed by the surgeon. Benefits are granted in accordance with the Swiss Federal Health Insurance Act. In Switzerland, a medical prescription for ambulatory physiotherapeutic treatment (AT) includes a maximum of 30 min of treatment by a trained physiotherapist two to three times a week for 9 sessions and medical treatment by a doctor when required.

A medically prescribed stay at a convalescence center (Cure) includes 1 to 2 therapy units of 30 min per day. The Swiss health insurance companies grant full payment for medical consultations, medication and therapies as prescribed. A daily contribution to lodging (€ 10) for a maximum of 21 days per year is paid by the mandatory basic insurance according to the Swiss Federal Health Insurance Act. Additional therapies, medical services and a higher contribution to lodging may be paid by an optional supplementary health care insurance or by the patient. During Cure, patients may lodge in a specialized convalescence hotel (health resort or spa hotel) or in a hotel close to a rehabilitation clinic, where they attend medical consultations and prescribed therapies. Compared to IR, Cure does not provide permanent medical care (24 h of permanent medical and nursing care) and cost for accommodation is not fully covered by the mandatory basic insurance.

For a more intense inhouse rehabilitation (IR in the following) in accordance with the Swiss Federal Health Insurance Act, the rehabilitation facility has to be qualified by the public health authority to provide musculoskeletal rehabilitation. If the requirement of medical care and rehabilitation for a patient was confirmed by the patient’s insurance company, commitment to cover costs is granted for 2 to 3 weeks based on a fixed daily rate, including medical and nursing services as well as full coverage of board and lodging. Therapeutic measures include 3 to 4 therapy sessions of different type according to the medical prescription of the medical doctor in charge: physical therapy, group therapies, thermal applications a.o.

### Measures

Socio-demographic and potentially confounding parameters, such as sex, age, living alone, smoking, alcohol consumption, sports, and formal education were recorded on a standardized form at admission to the hospital (T0) [15]. Comorbidities (excluding joint diseases) were recorded on the Self-administered Comorbidity Questionnaire (SCQ) and by review of the medical records [16].

Symptoms and activities were assessed using the WOMAC. This is a disease-specific multidimensional self-assessment instrument for osteoarthritis of the lower extremity measuring pain (5 items), stiffness (2 items) and physical functional ability (17 items) leg- but not joint-specifically [13]. All 24 items were evaluated on a continuous visual analogue scale ranging from ‘no symptoms/no limitation’ to ‘maximal symptoms/maximal limitation’. The validated German version was used [17]. According to the missing rule of the user’s guide, the subscores can only be determined if at least 4 of the 5 pain items, 1 of the 2 stiffness items, and 14 of the 17 function items are completed [13].

The Iowa Level of Assistance Scale (ILOAS) is a functional outcome and mobility instrument developed for patients after joint replacement [18]. It requires that five functional activity tests are performed in the following defined order: transfer from supine to sitting, stand up from the bed (transfer from sitting to standing), walk 4.57 m (15 ft), climb up and down three steps, walking speed over a distance of 13.4 m (44 ft). The first four tasks are graded according to the level of assistance required on a scale from 0 (independent) to 6 (not tested for safety reasons). The assistance device used in tasks that involve standing or mobilization is scaled from 0 (no assistance device) to 5 (walking frame or rollator). The ambulation velocity is rated on an ordinal scale from 0 (≤20 s) to 6 (>70 s). The total score is calculated and ranges from 0 (independence in all tasks and no assistive device required) to 50 (for medical reasons or safety reasons unable to perform any of the five tasks and using a walking frame or rollator as an assistive device). After total hip or knee replacement, assessing the functional outcome with ILOAS was reliable, valid and responsive [18, 19].

The “Timed Up and Go” (TUG) is a modified, timed version of the “Get-Up and Go” test first described by Mathias et al. [20]. It is a simple and inexpensive method to assess basic mobility with everyday movements [21]. Time is measured while the following basic functional mobility tasks are performed in the following sequence: getting up from a chair (seat height 46 cm) with arm rest, walk 3 m, turn around, walk back and sit down again [22]. The TUG was shown to be responsive in detecting improvements after arthroplasties in the early postoperative phase [23].

### Analyses

Primary outcome measure was the change in WOMAC score assessed at admission to the hospital (T0) prior to surgery and at follow-up at 6 months post-surgery (T2). For the use of the WOMAC, licensing processes and costs had to be paid based on the specific information about the research project. To reduce costs, the WOMAC was not administered at T1. Further assessment at T0 included sociodemographic data, comorbidities, TUG and ILOAS. Functional assessments, TUG and ILOAS, were measured for the second time after arthroplasty (T1) in the hospital (mean duration of stay: 9.7 days +/− 3.9). These measures of functional mobility were used to analyse a possible relation to the different rehabilitation settings. Since they were not used as outcome measures, TUG and ILOAS were only assessed at T0 and T1. At T2, the functional tests were not performed due to expenses and inconveniences for the patients for travelling back to the hospital for the examination. Follow-up at 6 months post-surgery included in addition to the WOMAC score the discharge destination after hospital and information about post-hospital treatment (T2).

All WOMAC scores were transformed into a scale ranging from 0 (“no symptoms/no limitation”) to 100 (“maximal symptoms/maximal limitation”) to facilitate comparison of the descriptive data [24]. Standardized effect sizes were calculated to obtain a quantification of the score changes or “effects” of the intervention [25]. Effect sizes (ES) of 0.00 – 0.19 reflect very small, 0.20–0.49 small, 0.50–0.79 moderate, and 0.80 or more large improvement [26]. A positive ES reflects a health improvement.

Statistical significance between the independent post-hospital treatment groups, functional mobility measures and WOMAC scores were analysed pairwise by the nonparametric Mann–Whitney-U- test and the Pearson Chi-square test. The comparison between two groups only was chosen in favor of more detailed statistical information about the differences between groups and to optimise interpretation of significant differences. The comorbidities were quasi continuous and were, therefore, analysed with the Wilcoxon signed-rank test. The level of statistical significance was set at the 95 % confidence level (*p* < 0.050).

All analyses were performed using the statistical software package SPSS 20.0 for Windows (SPSS Inc., Chicago, IL, USA).

## Results

### Patients

A total of 221 patients were recruited for this study from February to December 2010. 177 patients (80 %) were included to the study at the Cantonal Hospital of St. Gallen (KSSG) and 44 (20 %) at the Inselspital (ISB), Bern University Hospital in Bern, Switzerland. 109 patients (49 %) were operated at the knee unilaterally, 99 (45 %) at the hip unilaterally. All others were operated at the hips or knees bilaterally and one person had a combined operation of hip and knee. 20 patients (9 %) had to be excluded for reasons of non-compliance. Of the remaining 201 patients, the number of complete data sets is indicated in the tables.

Socio-demographic variables and disease-relevant characteristics as well as the characteristics of the different treatment groups are presented in Table 1. Significant differences were found between AT and Cure as well as between AT and IR in the categories age and living. The comorbidities differed significantly between the AT and IR group. Patients treated in an ambulatory, outpatient setting were significantly younger than the patients treated in Cure (*p* = 0.029) or IR (*p* = 0.001). Patients living with a partner or children were significantly more often referred to AT than to Cure (*p* = 0.043) or to IR (*p* = 0.033). The larger proportion of patients referred to Cure was female (67 %) whereas half of the patients in the AT group were male (*p* = 0.045).

**Table 1:** Patient’s demographic and clinical characteristics at entry to the hospital (T0)

		Ambulatory treatment (AT)	Cure	Inpatient rehabilitation (IR)	*p*	*p*	*p*
		*n* = 93 (46.3 %)	*n* = 46 (22.9 %)	*n* = 62 (30.8 %)	AT vs. Cure	AT vs. IR	Cure vs. IR
Age (y)		65 (+/− 11)	70 (+/− 9)	71 (+/− 8)	0.029*	0.001*	0.391
Sex (%)	Female	46 (50 %)	31 (67 %)	37 (60 %)	0.045*	0.212	0.412
Living (%)	Alone	16 (17 %)	15 (33 %)	20 (32 %)	0.043*	0.033*	0.969
	With partner or Children	76 (82 %)	31 (67 %)	42 (68 %)			
Smoking (%)	Yes	15 (16 %)	3 (7 %)	5 (8 %)	0.112	0.142	0.762
Alcohol consumption (%)	No	30 (32 %)	9 (20 %)	19 (31 %)	0.232	0.384	0.227
	Sometimes	48 (52 %)	30 (67 %)	31 (50 %)			
	Daily	12 (13 %)	6 (13 %)	12 (19 %)			
	Several times per day	3 (3 %)	0 (0 %)	0 (0 %)			
Sports (%)	None	35 (38 %)	12 (26 %)	21 (37 %)	0.286	0.108	0.384
	<1 h/week	15 (16 %)	5 (11 %)	4 (7 %)			
	1 – 2 h/week	22 (24 %)	13 (28 %)	10 (18 %)			
	>2 h/week	21 (23 %)	16 (35 %)	22 (39 %)			
Comorbidities (%)	None	35 (38 %)	19 (41 %)	16 (26 %)	0.962	0.022*	0.059
	1	34 (37 %)	11 (24 %)	15 (24 %)			
	2	10 (11 %)	12 (26 %)	19 (31 %)			
	>2	14 (14 %)	4 (9 %)	12 (19 %)			
Joint (%)	Hip unilateral	45 (49 %)	22 (48 %)	22 (35 %)	0.856**	0.188**	0.343**
	Knee unilateral	46 (49 %)	24 (52 %)	31 (50 %)			
	Hip bilateral	1 (1 %)	0 (0 %)	1 (2 %)			
	Knee bilateral	1 (1 %)	0 (0 %)	7 (11 %)			
	Hip and knee	0 (0 %)	0 (0 %)	1 (2 %)			
Education (%)	Basic school (8–9 years)	23 (25 %)	11 (24 %)	17 (27 %)	0.882	0.597	0.821
	Vocational training	48 (52 %)	25 (54 %)	34 (55 %)			
	College/high school	11 (12 %)	7 (15 %)	6 (12 %)			
	University	9 (10 %)	3 (7 %)	5 (7 %)			

Age is the arithmetic mean (+/− standard deviation), all other values are n with the corresponding % at T0 (=admission to hospital). *AT* ambulatory treatment, *IR* inpatient rehabilitation, h/week hours per week, *statistical significance with *p* < 0.050, **statistical calculation only with two groups: hip and knee (i.e., not specified whether uni- or bilateral)

All other categories of socio-demographic variables and disease-relevant characteristics between the three patient groups shown in Table 1 do not differ significantly between groups.

### Functional mobility measures

The functional mobility measures at admission to and before discharge from the hospital are shown in Table 2. In all rehabilitation groups, the time for the TUG and the ILOAS score increased from T0 at admission to hospital to T1 after arthroplasty. This means that the patients had, on average, a decrease in function in the first phase after their joint surgery. At admission to the hospital and before surgery (T0), the Cure group had the lowest TUG time and ILOAS score (reflecting the best function). After the operation, TUG time and ILOAS score were lowest in the AT group (27.5 s, 12.2 score points respectively), higher in the Cure group (33.9 s, 13.9 respectively) and highest in the IR group (40.0 s, 16.0 respectively).

**Table 2:** Functional mobility

	Ambulatory treatment			Cure			Inpatient rehabilitation			*p* at T0			*p* at T1		
	n	T0	T1	n	T0	T1	n	T0	T1	AT vs Cure	AT vs IR	Cure vs IR	AT vs Cure	AT vs IR	Cure vs IR
TUG (s)	89	11.7 (6.6)	27.5 (18.2)	39	10.8 (3.4)	33.9 (22.0)	52	12.0 (5.1)	40.0 (32.8)	0.910	0.040*	0.085	0.169	0.001*	0.252
ILOAS	87	0.8 (2.4)	12.2 (5.0)	34	0.4 (0.9)	13.9 (6.0)	48	1.5 (3.4)	16.0 (7.0)	0.494	0.202	0.133	0.102	0.000*	0.044*

All values are arithmetic mean (standard deviation). *n* number of complete data sets, *T0* admission to hospital, *T1* discharge from hospital (postoperative), *TUG* Timed Up and Go, *ILOAS* Iowa Level of Assistance Scale, *s* seconds, * statistical significance with *p* < 0.050

Significant differences were measured between AT and IR in both measures at T0 (TUG *p* = 0.040, ILOAS *p* = 0.020) and at T1 (TUG *p* = 0.001, ILOAS *p* < 0.001). All other calculations comparing the functional mobility measures at T0 and T1 were not statistically significant.

### WOMAC scores

WOMAC scores at admission and at follow-up as well as the statistical analysis are shown in Table 3. In general, independent of the post-hospital treatment destinations, the global score of WOMAC as well as in the subscales of pain, stiffness and function improved. The improvements ranged between ES = 0.95 (stiffness, AT) and ES = 1.87 (global score, Cure) reflecting large effects for all dimensions. The Cure group had the highest WOMAC scores in pain (47.9), stiffness (47.7) and function (49.4) as well as in the global WOMAC score (48.9) prior to the arthroplasty. After the operation, the same group showed the largest reductions in scores, respectively largest improvements with effect sizes from 1.35 (stiffness) to 1.87 (global score). The subscore of stiffness showed the smallest improvements in all rehabilitation groups when compared to the subscores of pain and function.

**Table 3:** WOMAC-scores

		Ambulatory treatment				Cure				Inpatient rehabilitation				*p* at T2		
	n	T0	T2	∆	ES	T0	T2	∆	ES	T0	T2	∆	ES	∆AT vs. ∆Cure	∆AT vs. ∆IR	∆Cure vs. ∆IR
Global	186	40.9 (19.5)	11.5 (9.5)	−29.4 (20.0)	1.51	48.9 (19.4)	12.7 (10.7)	−36.2 (19.3)	1.87	46.0 (21.2)	18.0 (16.0)	−28.0 (20.3)	1.32	0.037*	0.937	0.052
Pain	198	40.1 (21.2)	9.4 (9.6)	−30.7 (21.4)	1.45	47.9 (22.7)	8.9 (7.9)	−39.0 (23.0)	1.72	44.5 (20.3)	13.6 (16.7)	−30.9 (22.4)	1.53	0.036*	0.448	0.101
Stiffness	197	40.9 (28.1)	14.1 (16.4)	−26.7 (28.5)	0.95	47.7 (25.6)	13.0 (11.2)	−34.6 (26.4)	1.35	47.1 (27.8)	17.7 (21.0)	−29.4 (31.0)	1.06	0.149	0.496	0.605
Function	189	41.5 (20.8)	12.0 (10.6)	−29.5 (21.1)	1.41	49.4 (20.1)	13.7 (12.4)	−35.7 (20.1)	1.78	46.4 (22.9)	19.2 (17.4)	−27.2 (22.2)	1.19	0.067	0.838	0.059

All values are arithmetic mean (standard deviation). *WOMAC* Western Ontario and McMaster Universities Index ranging from 0 = no symptoms/no limitation to 100 = maximal symptoms/maximal limitation, *n* number of complete data sets, *T0* admission to hospital, *AT* ambulatory treatment, *IR* inpatient rehabilitation, *T2* 6 months follow-up, *∆* difference (T2-T0), *ES* effect size, * statistical significance with *p* < 0.050

There was no general difference in the health state of the three rehabilitation groups prior to hip or knee arthroplasty (data not shown). Prior to surgery (at T0), there was a statistically significant difference in the global score (*p* = 0.017) and the subscore of function (*p* = 0.022) between the AT and the Cure group. Postoperatively (T2), Cure patients showed significantly higher improvements in pain (*p* = 0.036) and in the global score (*p* = 0.037) of WOMAC than the AT group. All other dimensions (AT vs. Cure) and the other differences between AT and IR as well as Cure and IR, respectively, were not statistically significant.

### Associations of cofactors to disability at baseline

Disability at baseline (T0) was associated to mobility at baseline (TUG: explained variance 12.5 %, *p* < 0.001) and to number of comorbidities (explained variance 2.3 %, *p* = 0.027), but not to living with partner/alone (*p* = 0.262). However, the whole model showed low fit with explained variance of 14.7 %.

## Discussion

This natural, observational two-center study analysed the functional status and the socio-demographic, disease-relevant factors in patients before and immediately after hip or knee arthroplasty in relation to three different rehabilitation settings. The first hypothesis was confirmed, i.e. older patients with more comorbidities and preoperatively worse mobility status who were living alone were postoperatively more likely to be referred to an intensive inpatient rehabilitation clinic. The differences between IR and AT were largest and statistically significant. However, the differences in functional tests (TUG and ILOAS) before surgery between AT and Cure were small and not everywhere consistent. According to the results of this study, functional status at baseline currently seems to influence the decision where to refer the patient for rehabilitation and patients with the highest need of medical and social support were referred to most supportive locations. The tendency to a higher level of functional dependency of patients referred to IR was in accordance with the literature describing that patients referred to intensive inpatient rehabilitation are older, suffer from more comorbidities, are living alone and have worse postoperative functional status [27–30]. Our results showed rather weak associations of disability, mobility and comorbidities at baseline.

The second hypothesis could not be verified. Highest functional score changes were observed in Cure, followed by AR and IR. This means that high therapy intensity did not automatically lead to high functional improvements. This is in accordance with the conclusion that inpatient rehabilitation compared to home-based rehabilitation did not lead to a difference in pain or functional outcomes [31]. Taking into account that the Cure group had the highest disability scores before treatment, regression to the mean has to be considered as a possible cause of the observed changes.

Cure, a special characteristic of the Swiss health system, has not been included in the studies mentioned before [27–30]. In some countries, the difference of factors influencing the discharge destination after the acute hospital stay was explained by the unequal access to health services due to different health insurance status [32]. In Switzerland, all patients have equal access to the health care system based on the mandatory basic health insurance. Nevertheless, depending on the optional supplementary health care insurances, the fact that the patients may have to contribute an important part to the cost of a Cure may prevent postoperative admission to Cure in some cases.

A systematic review studied the influence of patient characteristics on the outcome of hip and knee arthroplasty in patients with osteoarthritis [33]. Although older age was related to worse outcome in terms of function, all groups benefited from joint arthroplasty, independent of age [33]. In this study, large improvements were measured in all WOMAC categories as well as in the WOMAC global score independent of the rehabilitation modalities and the factors influencing the referral to the more intensive IR. Internationally, more different modalities of post-hospital treatment exist than investigated in this study. Based on this study, the decision where to refer a patient after the acute hospital should not be based on the therapy intensities and the expected improvement of rehabilitation.

In the present study, the score changes of three different rehabilitation settings for hip and knee arthroplasties were compared. The high number of patients with only few drop-outs at follow-up is a strength of the study. Limitations for this study may arise by the naturalistic study design. The decision where to refer the patient after arthroplasty is not based on distinct criteria or guidelines but based on the subjective assessment of the orthopaedic surgeon in agreement with the patient and the willingness to pay of the insurance. This may cause different kinds of bias such as channelling bias and confounding as described in the introduction. While some potential confounders like comorbidities and age were measured, others like experience of the surgeon and type of insurance were not. Limited information about the exact number of therapies, kind of therapies as well as the content of therapies and the use of medication is a limitation of the study. The heterogeneity and variability of the treatment could not be considered. It is difficult to standardise the amount and intensity of treatment because in the setting of elderly and multimorbid subjects therapy is individually tailored rather than standardized. This leads to a high variability and heterogeneity of treatments and the corresponding score changes. No examiner-rated measures were available at follow-up to validate self-reported improvements as indicated by WOMAC.

## Conclusions

We conclude that severely impaired patients were admitted to the postoperative treatment with the highest therapy intensity, i.e. inpatient rehabilitation and Cure. Highest health improvements 6 months after arthroplasty were not necessarily associated with higher therapy intensity. Patients referred to intensive inpatient rehabilitation were older, had more comorbidities and the most impaired mobility which may have affected health-changes in the mid-term.

## Abbreviations

AT: Ambulatory treatment
Cure: Medically prescribed stay at a convalescence center
ES: Effect size
EULAR: European League against Rheumatism
ft: Feet
IR: Inpatient rehabilitation
ILOAS: Iowa Level of Assistance Scale
m: Meter
s: Second
SCQ: Self-administered Comorbidity Questionnaire
SF-36: Short form 36
TUG: Timed Up and Go
WOMAC: Western Ontario and McMaster Universities Osteoarthritis Index

## References

[1] Brady OH, Masri BA, Garbuz DS, Duncan CP (2000). Rheumatology: 10. Joint replacement of the hip and knee--when to refer and what to expect. CMAJ.

[2] Jordan KM, Arden NK, Doherty M, Bannwarth B, Bijlsma JWJ, Dieppe P (2003). EULAR Recommendations 2003: an evidence based approach to the management of knee osteoarthritis: Report of a Task Force of the Standing Committee for International Clinical Studies Including Therapeutic Trials (ESCISIT). Ann Rheum Dis.

[3] Zhang W, Doherty M, Arden N, Bannwarth B, Bijlsma J, Gunther K (2005). EULAR evidence based recommendations for the management of hip osteoarthritis: report of a task force of the EULAR Standing Committee for International Clinical Studies Including Therapeutics (ESCISIT). Ann Rheum Dis.

[4] Ethgen O, Bruyère O, Richy F, Dardennes C, Reginster JY (2004). Health-related quality of life in total hip and total knee arthroplasty. A qualitative and systematic review of the literature. J Bone Joint Surg Am.

[5] Jones CA, Voaklander DC, Johnston DW, Suarez-Almazor ME (2000). Health related quality of life outcomes after total hip and knee arthroplasties in a community based population. J Rheumatol.

[6] Shan L, Shan B, Graham D, Saxena A (2014). Total hip replacement: a systematic review and meta-analysis on mid-term quality of life. Osteoarthr Cartil.

[7] Shan L, Shan B, Suzuki A, Nouh F, Saxena A (2015). Intermediate and long-term quality of life after total knee replacement: a systematic review and meta-analysis. J Bone Joint Surg Am.

[8] Falbrede I, Widmer M, Kurtz S, Schneidmüller D, Dudda M, Röder C (2011). Utilization rates of lower extremity prostheses in Germany and Switzerland: a comparison of the years 2005–2008. Verwendungsraten von Prothesen der unteren Extremität in Deutschland und der Schweiz. Ein Vergleich der Jahre 2005–2008. Orthopade.

[9] OECD. Hip and knee replacement. In: OECD, Health at a Glance 2013: OECD Indicators. OECD Publishing. [http://dx.doi.org/10.1787/health_glance-2013-38-en]. Access date: 18.04.2014.

[10] Enloe LJ, Shields RK, Smith K, Leo K, Miller B (1996). Total hip and knee replacement treatment programs: a report using consensus. J Orthop Sports Phys Ther.

[11] Jones DL, Westby MD, Greidanus N, Johanson NA, Krebs DE, Robbins L (2005). Update on hip and knee arthroplasty: current state of evidence. Arthritis Rheum.

[12] Ontario HQ (2005). Physiotherapy rehabilitation after total knee or hip replacement: an evidence-based analysis. Ont Health Technol Assess Ser.

[13] Bellamy N (1995). WOMAC osteoarthritis index. A user’s guide.

[14] Angst F, Aeschlimann A, Stucki G (2001). Smallest detectable and minimal clinically important differences of rehabilitation intervention with their implications for required sample sizes using WOMAC and SF-36 quality of life measurement instruments in patients with osteoarthritis of the lower extremities. Arthritis Rheum.

[15] Angst F, Aeschlimann A, Steiner W, Stucki G (2001). Responsiveness of the WOMAC osteoarthritis index as compared with the SF-36 in patients with osteoarthritis of the legs undergoing a comprehensive rehabilitation intervention. Ann Rheum Dis.

[16] Sangha O, Stucki G, Liang MH, Fossel AH, Katz JN (2003). The self-administered comorbidity questionnaire: a new method to assess comorbidity for clinical and health services research. Arthritis Rheum.

[17] Stucki G, Meier D, Stucki S, Michel BA, Tyndall AG, Dick W (1996). Evaluation of German version of the WOMAC (Western Ontario and McMaster Universities) osteoarthritis index. Z Rheumatol.

[18] Shields RK, Enloe LJ, Evans RE, Smith KB, Steckel SD (1995). Reliability, validity, and responsiveness of functional tests in patients with total joint replacement. Phys Ther.

[19] Cantieni M, Leensen T, Knuesel O, Oesch P (2009). Is the ILOAS a suitable assessment tool for functional status evaluation of patients with total knee arthroplasty during inpatient rehabilitation? Reproducibility, validity and responsiveness of the ILOAS. Physioscience.

[20] Mathias S, Nayak USL, Isaacs B (1986). Balance in elderly patients: The “Get-up and Go” test. Arch Phys Med Rehabil.

[21] Nordin E, Rosendahl E, Lundin-Olsson L (2006). Timed “Up & Go” test: reliability in older people dependent in activities of daily living-focus on cognitive state. Phys Ther.

[22] Podsiadlo D, Richardson S (1991). The timed “Up & Go”: a test of basic functional mobility for frail elderly persons. J Am Geriatr Soc.

[23] Kennedy DM, Stratford PW, Wessel J, Gollish JD, Penney D (2005). Assessing stability and change of four performance measures: a longitudinal study evaluating outcome following total hip and knee arthroplasty. BMC Musculoskelet Disord.

[24] Angst F, Pap G, Mannion AF, Herren DB, Aeschlimann A, Schwyzer HK (2004). Comprehensive assessment of clinical outcome and quality of life after total shoulder arthroplasty. Usefulness and validity of subjective outcome measurement. Arthritis Rheum.

[25] Cohen J (1992). A power primer. Psychol Bull.

[26] Kazis LE, Anderson JJ, Meenan RF (1989). Effect sizes for interpreting changes in health status. Med Care.

[27] de Pablo P, Losina E, Phillips CB, Fossel AH, Mahomed N, Lingard EA (2004). Determinants of discharge destination following elective total hip replacement. Arthritis Rheum.

[28] Forrest G, Fuchs M, Gutierrez A, Girardy J (1998). Factors affecting length of stay and need for rehabilitation after hip and knee arthroplasty. J Arthroplasty.

[29] Forrest GP, Roque JM, Dawodu ST (1999). Decreasing length of stay after total joint arthroplasty: effect on referrals to rehabilitation units. Arch Phys Med Rehabil.

[30] Munin MC, Kwoh CK, Glynn N, Crossett L, Rubash HE (1995). Predicting discharge outcome after elective hip and knee arthroplasty. Am J Phys Med Rehabil.

[31] Mahomed NN, Davis AM, Hawker G, Badley E, Davey JR, Syed KA (2008). Inpatient compared with home-based rehabilitation following primary unilateral total hip or knee replacement: a randomized controlled trial. J Bone Joint Surg Am.

[32] Mahomed NN, Koo Seen Lin MJ, Levesque J, Lan S, Bogoch ER (2000). Determinants and outcomes of inpatient versus home based rehabilitation following elective hip and knee replacement. J Rheumatol.

[33] Santaguida PL, Hawker GA, Hudak PL, Glazier R, Mahomed NN, Kreder HJ (2008). Patient characteristics affecting the prognosis of total hip and knee joint arthroplasty: a systematic review. Can J Surg.

